# Molecular detection of hepatitis B virus genotype E with immune escape mutations in chronic hepatitis B patients on long-term antiviral therapy in Jos, Nigeria

**DOI:** 10.4102/ajlm.v11i1.1677

**Published:** 2022-10-18

**Authors:** Joseph Anejo-Okopi, Edith Okeke, Pantong M. Davwar, Chika Onwuamah, Harris Onywera, Patience Omaiye, Mary Duguru, Ocheme J. Okojokwu, Otobo I. Ujah, Bulus Jonathan, Chima A. George, Ramyil S. Crown, Fiyaktu B. Yakubu, Judith O. Sokei, Leona C. Okoli, Onyemocho Audu, Seth C. Inzaule, Isaac O. Abah, Patricia Agaba, Oche O. Agbaji, Atiene S. Sagay, Claudia Hawkins

**Affiliations:** 1 Department of Microbiology, University of Jos, Jos, Nigeria; 2 AIDS Prevention Initiative in Nigeria, Jos University Teaching Hospital, Jos, Nigeria; 3 Department of Internal Medicine, University of Jos, Jos University Teaching Hospital, Jos, Nigeria; 4 Center for Human Virology and Genomics Nigeria Institute of Medical Research, Lagos, Nigeria; 5 Institute of Infectious Disease and Molecular Medicine, University of Cape Town, Cape Town, South Africa; 6 Division of Medical Virology, Department of Pathology, University of Cape Town, Cape Town, South Africa; 7 Research, Innovations, and Academics Unit, Tunacare Services Health Providers Limited, Nairobi, Kenya; 8 Department of Community and Family Health, College of Public Health, University of South Florida, Tampa, Florida, United States; 9 Department of Family Medicine, Plateau State Specialist Hospital, Jos, Nigeria; 10 Department of Family Medicine, Bingham University Teaching Hospital, Jos, Nigeria; 11 Department of Medical Microbiology and Parasitology, Bingham University Teaching Hospital, Jos, Nigeria; 12 Department of Chemical Pathology, Jos University Teaching Hospital, Jos, Nigeria; 13 Department of Epidemiology and Community Health, Benue State University, Makurdi, Nigeria; 14 Department of HIV and Global Hepatitis Program, World Health Organization, Geneva, Switzerland; 15 Department of Pharmacology, University of Jos, Jos University Teaching Hospital, Jos, Nigeria; 16 Department of Family Medicine, University of Jos, Jos University Teaching Hospital, Jos, Nigeria; 17 Department of Obstetrics and Gynaecology, University of Jos, Jos University Teaching Hospital, Jos, Nigeria; 18 Department of Medicine, Feinberg School of Medicine, Northwestern University, Chicago, Illinois, United States

**Keywords:** hepatitis B virus (HBV), genotyping, antiviral drug resistance, chronic hepatitis B

## Abstract

**Background:**

Previous studies in Nigeria have reported the presence of hepatitis B virus (HBV) genotype E and the availability of immune escape mutants. There is a paucity of data on chronic patients on long-term antiviral therapy for HBV infection.

**Objective:**

This study assessed HBV genotypes and drug resistance variants among patients with chronic HBV infection receiving tenofovir in Jos, Nigeria.

**Methods:**

This cross-sectional study consecutively enrolled 101 patients (51 with HIV/HBV co-infection and 50 with HBV infection only) on antiviral therapy from February 2018 to May 2019 at four hospitals in Jos, Nigeria. DNA quantification of HBV was performed on all samples; 30 samples with detectable viral load were selected for genotyping using Sanger sequencing by targeting the full-length sequences of reverse transcriptase gene of the HBV genome. Phylogenetic analysis was performed with reference sequences from GenBank. Escape mutant and drug resistance analysis were performed using HBV drug resistance interpretation and Geno2pheno.

**Results:**

Only 30 (29.7%) of the 101 study participants had detectable HBV DNA. Of these, six (20.0%) isolates were successfully amplified and sequenced. The identified genotype was E, including escape mutations L127R (16.7%) and G145A (16.7%).

**Conclusion:**

This study revealed exclusive dominance of genotype E in Nigeria. The S gene mutations G145A and L271R are known to be associated with modified antigenicity and impaired serologic assays, which may cause false negatives in the detection of anti-HBV surface antigen. The presence of mutants that are associated with vaccine immune escape may also have diagnostic and vaccine immune response implications.

## Introduction

Hepatitis B virus (HBV) infection is a public health problem worldwide, with an estimated global infection of 2 billion, 257 million of whom are chronically infected.^1,2^ Hepatitis B virus infection causes about 880 000 deaths yearly due to cirrhosis and hepatocellular carcinoma (HCC).^3^ The prevalence of HCC is rapidly increasing with most cases being attributable to chronic hepatitis B. The disease is endemic in sub-Saharan Africa, affecting more than 8.0% of the population, most of whom are infected in early childhood.^3^ Individual infections may resolve, become sub-clinical, chronic or result in liver damage. Sub-Saharan Africa has one of the highest HBV-related liver cancer rates in the world.^4,5^ Hepatitis B virus infection increases the risk of developing liver cirrhosis and HCC by 25.0% to 40.0%.^6^ Hepatitis B virus prevalence in Nigeria varies among different populations (ranging between 9.2% and 18.0%), including HBV/HIV co-infected population.^7,8,9^

There is mounting evidence for heterogeneity of HBV treatment outcomes and vaccination efficacy attributed to the variations in the distribution of HBV genotypes.^10^ Hepatitis B virus has been characterised into 10 genotypes: A–I^4,11^ and J which was recently identified in a single case in Japan.^12^ Genotypes A, D and E circulate in Africa, with the latter genotype prevailing in western Africa,^4,13^ including Nigeria.^14,15,16,17,18^ Looking at Nigeria’s huge population and cross-border movement, it is important that the molecular characterisation of HBV genotypes and prevalence of drug resistance mutations is determined for better management and epidemiological purposes. The investigation of HBV genotype is becoming more important due to its role in the pathogenesis and treatment response in both HBV-mono and HBV/HIV co-infected patients and the wide availability of antiviral therapy.^19^ Equally, drug resistance is a major clinical concern impacting clinical outcomes. Studies have reported the impact of some mutations that showed significant role of viral persistence on liver disease, immune escape and resistance to antiviral therapy.^20,21^

The reverse transcriptase encoded by the pol gene lacks effective proofreading ability, which has implications in HBV therapy.^22^ However, the use of low genetic barrier drugs such as lamivudine has contributed to the development of a drug-associated vaccine-escape mutant which is becoming a growing health concern. This mutant alters envelope antigen that permits the virus to escape the neutralisation antibody to hepatitis B surface antigen.^21^ This called for the need of drugs with higher genetic barriers to resistance than lamivudine or telbivudine.^19^ However, there is a paucity of knowledge regarding the HBV genotypes and resistant strains in chronic adult patients on long-term antiviral therapy. We therefore characterised HBV in chronic patients on tenofovir to identify circulating genotypes and drug resistance variants in Nigeria.

## Methods

### Ethical considerations

The study protocol was approved by the four study sites research ethics committees, namely: Jos University Teaching Hospital, Faith Alive Foundation, Bingham University Teaching Hospital and Plateau State Specialist Hospital (study approval numbers: JUTH/ADM/11/0517, FA/0112F, BUTH/EC/01-25-18B and PLASHREC/12018). After explaining the study objectives and procedures to the participants, a written informed consent was obtained from each patient before data and sample collection. The patients’ data were coded and reported without divulging personal health information. All other aspects of the study were conducted in accordance with the ethical standards of the Helsinki Declaration.

### Study design and population

We conducted a cross-sectional study between February 2018 and May 2019 at Jos University Teaching Hospital, Plateau State Specialist Hospital, Faith Alive Foundation and Bingham University Teaching Hospital, all in Jos metropolis, Nigeria. The 101 consecutively enrolled adult patients, including 51 patients co-infected with HIV, were confirmed to have chronic HBV infection after testing positive for hepatitus B surface antigen antibodies. For HBV sero-diagnosis, 5 mL of blood sample was collected, centrifuged at 2500 revolutions per minute for 1 min, and 1 mL of plasma was used for testing HBsAg by third-generation enzyme-linked immunosorbent assay (Monolisa, Bio-Rad, Paris, France) according to manufacturer’s instruction; a positive HBsAg result indicates current infection. All participants were on antiviral therapy (HBV/HIV: lamivudine/tenofovir combination, HBV-mono: tenofovir alone) ≥ 12 months.

### Sample preparation

The 5 mL of blood samples collected were centrifuged, and the sera separated, aliquoted, and stored at –80 °C until use. The HBV DNA detection and quantification were performed using automated COBAS^®^ AmpliPrep TaqMan HBV Test version 2.0 (Roche Diagnostics International AG, Rotkreuz, Switzerland). A total of 30 samples with detectable viral load were selected for DNA sequencing reactions.

### Hepatitis B virus DNA extraction

DNA extraction, amplification and direct sequencing of the full-length HBsAg and the reverse transcriptase genes were performed at Center for Human Virology and Genomics, Nigeria Institute of Medical Research, Lagos, Nigeria. Viral DNA was extracted from 200 µL of plasma samples using the ZR Viral DNA kit^TM^ (Zymo Research, Irvine, California, United States), according to the manufacturer’s protocol.

### Primers and nested polymerase chain reaction

Universal primers from previous study were used for detection of HBV genotypes,^23^ which were synthesised by Inqaba Biotech (Pretoria, South Africa). Two outer primer sets were used. Primer set 251F (5ʹ-GGA TGT GTC TGC GGC GTT T-3ʹ) and 1797R (5ʹ-GAC CCA CAA TTC KTT GAC ATA CTT TCC-3ʹ) were used for the initial amplification, and 251F (5ʹ-CGA ACC ACT GAA CAA ATG GC-3ʹ) and 1190R (5ʹ-TCA CCA TAT TCT TGG GAA CAA GA-3ʹ) used for the second amplification, as previously described.^23^ These primer sets were designed to generate overlapping fragments, and polymerase chain reaction (PCR) was performed using Platinum™ SuperFi™ PCR Master Mix (Thermofisher, Waltham, Massachusetts, United States). Each 25.0 µL reaction mixture contained 12.5 µL of 2× premix, 1.25 µL of each 10.0 µM primer, 5.0 µL of PCR water and 5.0 µL DNA template for first PCR while for the second PCR, 3.0 µL of first PCR product was used. Both PCR were carried out in Applied Biosystems MiniAmp Plus Thermal Cycler (ThermoFisher, Waltham, Massachusetts, United States) with the following cycling conditions: 95 °C for 3 min followed by 35 cycles of 97 °C for 30 s, 54 °C for 60 s and 72 °C for 60 s for the first round of PCR while 31 cycles were done for the second round of PCR. Each round of PCR was followed by a final extension of 72 °C for 10 min. Amplified products were verified using 1% agarose gel stained with GelRed^®^ (Biotium, Fremont, California, United States) on a Cyfox Gel Documentation System (Sysmex-Partec, Münster, Germany). The amplified products were purified using ExoSAP-IT (Thermofisher, Waltham, Massachusetts, United States) and quantified using dsDNA high-sensitivity (Thermofisher, Waltham, Massachusetts, United States) reagent on the Qubit-4^TM^ (Thermofisher, Waltham, Massachusetts, United States). Samples with DNA concentration ≥ 5 ng/µL and gel bands of appropriate size were selected for further processing.

### Purification and Sanger sequencing

Verified nested PCR products were purified using ExoSAP-IT^TM^ PCR Product Cleanup reagent (Thermofisher, Waltham, Massachusetts, United States) at 37 °C for 15 min (to digest excess primers and deoxynucleoside triphosphates), and 80 °C for 15 min (to inactivate the enzymes) according to manufacturer’s protocol. The sequencing reaction mixture contained 1.0 µL of BigDye® Terminator version 3.1 Ready Reaction Mix (Applied Biosystems, Foster City, California, United States), 4.0 µL of 5× sequencing buffer, 2.0 µL of template, 11.0 µL of double distilled water, and 2.0 µL of 10.0 µM 251F and 1190R primers. Cycle sequencing was performed using 251F and 1190R primers in a MiniAmp Plus Thermal Cycler with the following program: 25 cycles of 96 °C for 10 s, 50 °C for 5 s and 60 °C for 4 min. The cycle sequencing product was purified using the BigDye Xterminator® Solution (Applied Biosystems, Waltham, Massachusetts, United States) followed by the capillary electrophoresis on the 3130XL Genetic Analyzer using a 50 cm capillary array, and POP-7 polymer (Applied Biosystems, Waltham, Massachusetts, United States). All the generated sequences were analysed and assembled using CLC Genomic Workbench version 8.0.3 (CLC Bio, Aarhus, Denmark) and then subjected to NCBI nucleotide BLAST (https://blast.ncbi.nlm.nih.gov/Blast.cgi) for quality check. In this study, a chromatogram was considered of good quality if (1) at least 80% of total amplicon length was sequenced and (2) the noise-to-signal ratio was estimated to be < 5%.

### Phylogenetic analysis of hepatitis B virus sequences

To infer the evolutionary relationship between the six HBV nucleotide sequences in our study and HBV sequences from different parts of the world, we used Molecular Evolutionary Genetics Analysis (MEGA) X version 10.1.7 (Mega Software, Dortmund, Germany).^24^ First, a total 102 sequences (including a sequence from a chimpanzee) representing the HBV genotypes from different parts of the world were mined from the NCBI GenBank nucleotide database (https://www.ncbi.nlm.nih.gov/nucleotide/). The sequence from the chimpanzee was used as an out-group. The generated nucleotide sequence data were deposited in the GenBank database under the accession numbers ON236588–ON236593.

These sequences, together with the six from our cohort, were aligned using MUltiple Sequence Comparison by Log-Expectation (MUSCLE) software,^25^ which is a sequence alignment option in MEGA. The multiple sequence alignment was trimmed to 846-base pairs, followed by determination of the DNA model that best describes the nucleotide substitution pattern. This was performed using the maximum likelihood (ML) statistical method. The General Time Reversible (GTR) model with discrete Gamma distribution (*+G* = 0.51, with 5 rate categories) and some invariant evolutionary sites (*+I* = 35.51% sites) was chosen as the best model, based on its low Bayesian information criterion score (18 980). For the phylogeny reconstruction the ML statistical method was used with a bootstrap of 1000 replicates and the sub tree-Pruning-Regrafting (Extensive; SPR level 5) as the ML heuristic method. Moreover, we used the option of ‘moderate branch length filter’ to enable a semi-stringent exhaustive optimisation of the branch lengths of the phylogenetic trees. Initial trees for the heuristic search were generated using Neighbor-Joining and BioNJ algorithms and the phylogenetic tree with superior log likelihood value selected. Phylogenetic trees were produced in Newick format employing interactive Tree of Life interface (https://itol.embl.de/tree/).

To determine the genetic diversity of the six HBV sequences, we performed an alignment analysis using MEGA.^24^ The overall mean (average) and maximum distance were used as proxies for genetic diversity.

### Assessment of hepatitis B virus drug resistance mutations

The obtained sequences of overlapping surface (S) and pol gene were translated to the protein sequences and aligned with the references in BioEdit version 7.1.3.0.^26^ Escape mutant analysis and drug resistance analysis were performed using HBV Drug Resistance Interpretation and Geno2pheno (HBV) version 2.0 (https://hbv.geno2pheno.org/index.php). It is a program that searches for homology between the input sequence and other DNA sequences in the existing stored database for drug resistance and surface gene mutations. Genotype assignments were confirmed using the GenBank BLAST and HBVseq tools from the HIV Drug Resistance Database.^26,27^

### Statistical analysis

The obtained data were analysed using Statistical Package for Social Science software (version 19.0; IBM Corp., Armonk, New York, United States) and expressed as medians (with interquartile ranges [IQR]) for continuous variables, and as counts and percentages for discrete variables. Chi-squared test was used for discrete data.

## Results

Thirty (29.7%) of the 101 had detectable HBV DNA. Of the 30 patients, 11 (36.7%) were co-infected with HIV while 19 (63.3%) had HBV-mono infection. Seventeen (56.7%) were female; the mean age was 41 years. The proportion of patients with HBV DNA copies/mL of < 20 copies/mL was 22/30 (73.3%); 6/19 (31.6%) of HBV-mono and 2/11 (18.2%) of HIV/HBV had 20 copies/mL – 20 000 copies/mL. Median aspartate aminotransferase U/L was 28 (23.8–36.0) for HIV/HBV co-infected patients and 27 (IQR: 19–32) for those with HBV-mono infection. Median alanine aminotransferase was 26.1 (IQR: 19.8–35.5) for those with HIV/HBV dual infection and 27 (IQR: 20–41) for those with HBV-mono infection while platelet level was 259 (IQR: 198.3–298.8) for those with HIV/HBV dual infection and 195 (168–257) for those with HBV-mono infection.

### Phylogeny and genetic diversity of the hepatitis B virus sequences

**FIGURE 1:**
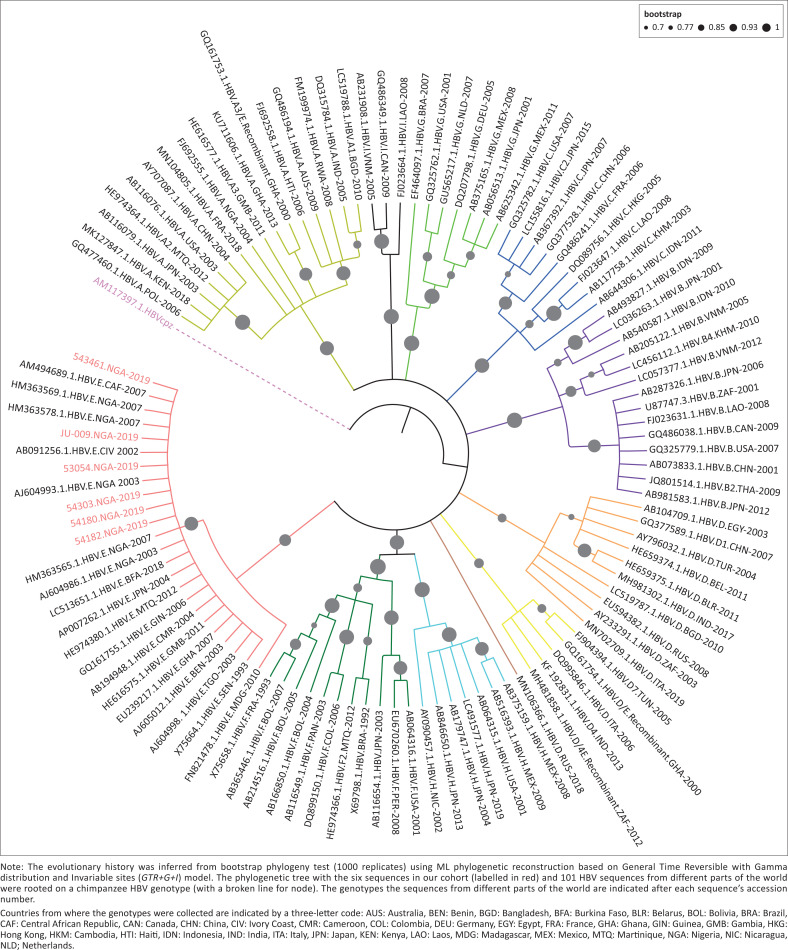
Phylogenetic maximum likelihood circular consensus tree with branch node statistics as viewed using interactive Tree of Life (https://itol.embl.de/tree/).

All the generated sequences were from the HBV-mono infected patients. The genotypes of the six HBV nucleotide sequences were inferred from the phylogenetic tree (Figure 1), which showed that all the sequences were HBV genotype E. All the six sequences clustered with HBV genotype E, most of which were from the West African region.

### Hepatitis B virus drug resistance report

According to the Geno2pheno (HBV) 2.0 report, all the six HBV genotype E sequences were susceptible to lamivudine, adefovir, entecavir, tenofovir disoproxil fumarate, and telbivudine. Two (33.3%) of the six genotypes, all from HBV-mono infected individuals, had escape mutations, the SH2 domain-containing adapter protein B protein of the surface-gene. One sample had L127R escape mutation while the other had G145A at a position first described as vaccine escape mutation. These mutations are amino acid substitutions within the major hydrophilic region called the ‘a’ determinant (124–147).

## Discussion

Our findings showed a predominance of genotype E consistent with previous studies from Nigeria and other West African regions.^4,14,16,17,18,28,29,30^ We however found that the genotypes had a lower diversity compared to a previous report,^14^ although other studies have equally observed a low genetic diversity of HBV genotype E in West Africa.^18,31^ A possible explanation may be due to natural selection, drug pressure and hyperendemicity of HBV genotype E infection in other West African countries.^28^

The ease of transmission and epidemiological pattern of HBV have been linked to genotype variability, as well as clinical virological parameters. The pathogenicity of HBV has been shown to vary with genotype and certain mutations have been associated with genotype diversity that influences disease outcome, HCC development, diagnostic testing and treatment outcomes.^28^ Studies have shown a higher severity of liver disease progression and HCC in patients infected with C and D genotypes than those infected with A or B genotypes.^32,33^ Although the clinical implications of genotype E, the predominant genotype in our study, have not been well reported, it is speculated that patients with genotype E have better liver disease prognosis than other genotypes.^29^ Larger studies are needed to provide further evidence on good clinical prognosis for patients infected with genotype E. Nonetheless, these findings suggest the need for adoption of individualised genotypic testing, which may play a key role in guiding future treatment strategies to combat the HBV disease. In the interim, early treatment is warranted to ensure good HBV prognosis.

As reported in other studies, we did not find any tenofovir or underlying lamivudine-associated mutations including the rtA194T that was earlier reported to be resistant to tenofovir.^34,35^ This suggests that tenofovir-containing and monotherapy regimen is still effective against the virus in our setting. However, we observed vaccine escape mutations (L127R and sG145A) in two (33.3%) of the six genotypes. The finding of sG145A is consistent with findings from Nigeria,^16^ Ghana^36^ and Gabon.^37^ These HBsAg vaccine escape mutants may have arisen as a result of specific selection such as the host immune system due to vaccination or antiviral selective pressure that truncates the production of HBsAg resulting in low plasma HBV DNA levels.^38^ Although we did not take the history of patients’ vaccination, but all the patients were on antiviral therapy (≥ 12 months), including mono-lamivudine or lamivudine-containing regimen before switching to tenofovir, and this may suggest a possible reason for emergence of drug-associated potential vaccine-escape mutants (sG145A) in the study patients.

Similarly, mutations arising in the S ORF region because of inherent or selective pressure by antiviral drugs can end up in the emergence of virus escape mutants in the adjacent S region with subsequent lack of response to HBV vaccine. The presence of this mutant over time could lead to partial replicative capacity of resistant HBV variant, and lack of response to HBV vaccine.^39^ This mutation may be due to poor adherence to antiviral therapy and the use of drugs with a low genetic barrier to resistance such as lamivudine, which was the major drug used in Nigeria before switching to tenofovir among HBV-mono infected patients. This mutation sG145 confers immune escape competence on the virus,^40,41^ which has negative impact on humoral immune response to HBV vaccine. However, a recent study has shown that the risk of transmission of HBV infection is low in a vaccinated individual.^42^ Similarly, the presence of this mutation in immune-compromised patients could be responsible for HBV reactivation among persons previous immune to hepatitus B surface antigen antibodies, suggesting immune escape mutant.^43^ Also, this mutation among others can be responsible for virus detection failure in most routine screening tests.^44^ This is because an assay used for HBV screening may give false-negative results if the test kit was not designed to detect mutants in the ‘a’ determinant. This is common in the clinical setting where the patients have HBsAg negative result, yet will have classical symptoms and, if tested for HBV DNA, will have positive high HBV DNA result. The detection of some of these emerging mutants has become a major challenge to commercially available immunoassays.

The L127R escape mutation in the ‘a’ determinant region observed in our study has been associated with HBV antigenicity, diagnostics and immunogenicity.^45^ Overall, L127R mutation was mostly associated with patients who had experienced lamivudine treatment in other African countries.^36,37^ The long-term impact of HBsAg vaccine escape mutations on the natural history of chronic HBV remains unclear. However, it has been hypothesised that the presence of stop codons may favour the production of a truncated HBsAg (despite low serum HBV DNA), which accumulates in the endoplasmic reticulum and induces oxidative stress to enhance cell proliferation.^38^ This may also explain the transmission of HBV genotype E infection even in vaccinated individuals.

Studies on the HBV genotypes, immune escape and antiviral drug resistance mutations in Nigeria are scarce. This is the first published study, to the best of our knowledge, that characterised HBV genotypes and drug resistance in chronic hepatitis B patients (HBV/HIV co-infected and HBV-mono infected patients) on long-term antiviral therapy (tenofovir) in Nigeria. However, one recent study assessed the effect of HBV mutations with liver severity in HIV/HBV co-infected antiretroviral therapy-naïve patients in Nigeria.^27^ In this study, HBV mutations were independently associated with liver disease severity, but the effect declined after antiretroviral therapy initiation. Our study however, highlighted the need to assess HBV mutations due to the potential link with disease severity.

### Limitations

There are limitations to this study that need to be highlighted. It is a cross-sectional study with a small sample size, and low amplification and sequencing rate, yielding only 20.0% of the intended samples. Many factors could be responsible for this. For example, several freeze-thawing cycles and poor storage due to frequent power outage could have affected the sample integrity, resulting in decreased HBV DNA quantity. Other causes include intrinsic reagent-related issues and impact of HBV/E on the sensitivity of HBsAg assays due to surface antigen gene mutations located outside the sequenced region.^46^ Also, there is a growing concern that the accumulation of escape mutations may lead to detection failure of HBsAg using the available commercial diagnostic assays. This in turn may lead to a false-negative HBV result. Moreover, this has an impact on risk of transmission, including rendering the vaccination ineffective due to undiagnosed mutant carriers.

### Conclusion

Our study confirmed the dominance of genotype E, and it has great public health significance, given that the protective vaccine currently in use by the national vaccination programme is from the HBV-A2 strain. Additionally, no patients had HBV drug resistant mutations, suggesting that the use of tenofovir is an effective treatment in the management of HBV infection in both chronic HBV/HIV co-infected and HBV-mono infected patients. The study also showed the presence of mutation associated with immune escape mutant in chronic HBV-infected patients on long-term antiviral therapy. Therefore, further larger study is required to understand the dynamics of immune escape mutant on HBV diagnosis, and impact of genotypes on treatment outcomes.
